# Behavioral alterations induced by repeated saxitoxin exposure in drinking water

**DOI:** 10.1186/s40409-016-0072-9

**Published:** 2016-05-17

**Authors:** Felipe Diehl, Patricia Baptista Ramos, Juliane Marques dos Santos, Daniela Martí Barros, João Sarkis Yunes

**Affiliations:** Postgraduate Program in Physical, Chemical and Geological Oceanography, Institute of Oceanography, Federal University of Rio Grande (FURG), Rio Grande, RS Brazil; Postgraduate Program in Compared Animal Physiology, Institute of Biological Sciences, Federal University of Rio Grande (FURG), Rio Grande, RS Brazil; Institute of Oceanography, Federal University of Rio Grande (FURG), Caixa Postal 474, Rio Grande, RS CEP 96203-900 Brazil

**Keywords:** Saxitoxin, Drinking water, Behavior, Aversive memory, Spatial memory, Rats

## Abstract

**Background:**

Blooms of the saxitoxin-producing cyanobacterium *Cylindrospermopsis raciborskii* have been contaminating drinking water reservoirs in Brazil for many years. Although acute effects of saxitoxin intoxication are well known, chronic deleterious outcomes caused by repeated saxitoxin exposure still require further investigation. The aim of the present work is to investigate the effects of consumption of drinking water contaminated with *C. raciborskii* for 30 days on learning and memory processes in rats.

**Methods:**

The effects of saxitoxin (3 or 9 μg/L STX equivalents) or cyanobacteria on behavior was determined using the open field habituation task, elevated plus maze anxiety model task, inhibitory avoidance task, and referential Morris water maze task.

**Results:**

No effects of saxitoxin consumption was observed on anxiety and motor exploratory parameters in the elevated plus maze and open field habituation tasks, respectively. However, groups treated with 9 μg/L STX equivalents displayed a decreased memory performance in the inhibitory avoidance and Morris water maze tasks.

**Conclusions:**

These results suggest an amnesic effect of saxitoxin on aversive and spatial memories.

## Background

Paralytic shellfish toxins (PST) constitute a group of neurotoxins produced by marine dinoflagellates and freshwater cyanobacteria, among them, saxitoxin (STX) represent the more frequent and toxic component. Overgrowth of dinoflagellates in the sea causes a toxic event commonly known as “red tide”, which is often associated with massive death of fish, bird, and other marine animals [1]. More than 30 structurally related naturally occurring PST molecules have been described, with all containing two guanidinium moieties [2–6]. Although its basic molecular skeleton comprises a 3,4,5-triakyl tetrahydropurine grouping, the toxin can be divided into the following three classes depending on the side-chain chemical structure: N-sulfocarbamoyl, decarbamoyl, and carbamate. The main species responsible for contamination in marine waters are three morphologically distinct genera of dinoflagellates (*Alexandrium* sp., *Pyrodinium* sp., and *Gymnodinium* sp.), while four different species of cyanobacteria (*Anabaena circinalis*, *Aphanizomenon flos-aquae*, *Cylindrospermopsis raciborskii*, and *Lyngbya wollei*) are the main species responsible in freshwaters [1–7].

An increase has been observed in the blooms of toxic STX-producing cyanobacteria in eutrophic water bodies worldwide [8, 9]. Cyanobacterial STX has attracted much scientific and public attention due to their toxicity and extensive geographic distribution [10–15]. High concentrations of PST (1.57 μg/L STX equivalents) are detected in freshwater reservoirs in Brazil [16]. Therefore, the guideline concentration for STX in drinking water was described as 3.0 μg/L STX equivalents in the regulation number 2914/2011 of the Brazilian Ministry of Health. The same STX concentration was calculated and adopted as a health alert for STX equivalents in Australia [17]. However, there is no data concerning the toxicological effects of repeated consumption of contaminated drinking water at this STX concentration.

PST accumulates in marine shellfish and freshwater mussels at a very high concentration as a result of filter-feeding of dinoflagellates and cyanobacteria [18]. Subsequent human consumption of PST-contaminated shellfish and mussels commonly leads to the development of paralytic shellfish poisoning (PSP) with significant illness and mortality [6]. Symptoms of PSP intoxication include paresthesia of the mouth and lips, immobilization of the tongue, asthenia, dystonia, ataxia, dyspnea, nausea, vomiting, hypotension, tachycardia, and muscular and upper extremity weakness, followed by death in severe cases as a result of respiratory arrest [6, 19]. Relatively few studies have reported the poisoning of STX via water contamination [9].

Although acute effects of PST intoxication are well known, chronic deleterious effects on humans and other animals require further research. In addition to these diverse systemic effects, major toxic effects of STX are found in the brain [7]. STX is thought to be capable of crossing the blood–brain barrier (BBB), as it accumulates in the central nervous system (brain and medulla oblongata) [7, 20]. However, the transport mechanism through the BBB is still unknown.

The molecular action mechanism of STX is to block depolarization of excitable cells by reversibly binding to the voltage-gated sodium channels in the brain, peripheral nervous system, and muscles [4, 21]. The effects on other channels including HERG K1 channels and L-type calcium channels have also been reported [22, 23].

Although the primary molecular targets for STX in the CNS have been well studied, there is increasing evidence that the mode of action of PSPs extends beyond inhibition of neuronal transmission. It was demonstrated that exposure to STX from *Aphanizomenon flos-aquae* causes cell death in zebrafish brain tissue, with low doses inducing apoptosis and higher doses inducing necrosis [6]. Intraperitoneal (i.p.) administration of STX in some discrete rat brain regions has been reported to induce significant changes in quantities of neuroactive amino acids and serotonin [24]. However, i.p. administration of a sublethal dose of STX in rats resulted in acute alteration in dopamine production and its metabolite 3,4-dihydroxyphenylacetic acid (DOPAC) [25]. These and many others neurophysiological aspects are important to modulate memory processes like formation and consolidation of long-term memory (LTM).

Memory formation process is susceptible to interference of many external agents and involves many biochemical and molecular events in specific brain regions, like hippocampus, amygdala and neocortex [26]. Moreover, the hippocampus has been identified as a major target site for triggering factors of damage, such as disease processes or neurotoxic exposure [27].

Tetrodotoxin (TTX) and lidocaine are widely used drugs in behavioral studies to transiently inactivate brain structures [28]. Both are specific voltage-gated sodium channels blockers, and thus prevent impulse generation and conduction, similar to the mechanism of STX [29]. Several reports have proved that hippocampal and amygdalar inactivation by TTX and lidocaine impairs several types of memories, including aversive contextual and spatial memories, but little is known about the effects of STX on cognition [28, 30, 31].

The present study investigated the effect of repeated exposure to water contaminated with PST-producing cyanobacteria at concentrations of 3 or 9 μg/L STX equivalents on learning and memory processes using rat memory behavioral models.

## Methods

### Source of toxins

**Fig. 1:**
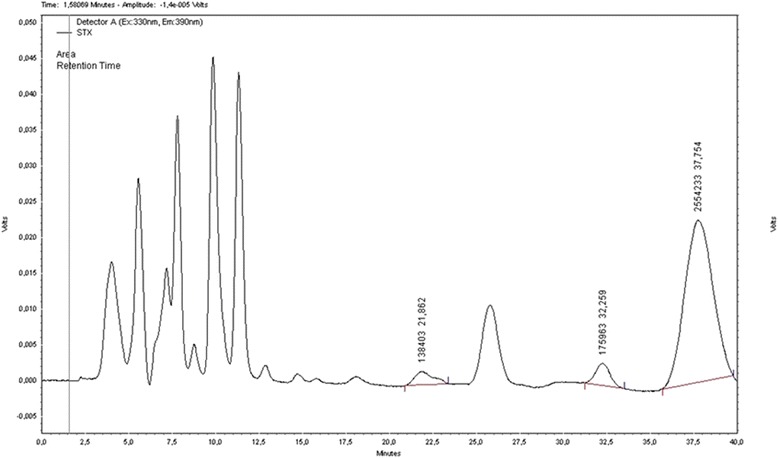
Chromatogram showing PST from *C. raciborskii* used in the experiments. The toxin peak levels separated at 21.8, 32.2 and 37.7 min correspond to neosaxitoxin, dc-saxitoxin, and saxitoxin, respectively

Cells of *C. raciborskii* from the FCL/FURG culture collection were cultured in ASM-1 medium at 25 ± 1 °C and employed as a source of STX [32]. PST produced by *C. raciborskii* was detected by HPLC/FLD with a major (STX) and two minor peaks (neosaxitoxin and dc-saxitoxin) (Fig. 1), which were analyzed and compared with the commercial standards of STX, neosaxitoxin, and dc-saxitoxin (NRC, Canada). This strain was also examined for microcystin and cylindrospermopsin production by using of specific ELISA kits. No positive results were obtained within the kits detection range (0.05–2.5 μg/L).

A culture of the cyanobacteria *Aphanothece* sp. was used as a negative control for an unspecific cyanobacteria effect in the behavioral experiments. This strain of *Aphanothece* sp. did not produce toxins such as microcystin, cylindrospermopsin or PST and was cultured in BGN/2 medium (modified from the original BG11 composition by adding only half of the total nitrate composition) [33, 34].

### Animals

Female Wistar rats (2 to 3 months old, weighting 210 to 300 g, *n* = 120) from our breeding facility were used. Animals were housed in plastic cages (five per cage) and maintained under a 12-h light/dark cycle and at a constant temperature of 24 ± 1 °C. The rats had access to water and food ad libitum.

Experiments with rats were performed in strict accordance with the Brazilian law and the Brazilian College of Animal Experimentation (COBEA), and were approved by the Ethics Committee of FURG (process number P025/2011 CEUA/FURG).

### Experimental design

Experimental animals received water artificially contaminated with cyanobacterial cultures or its growth medium (control groups) for 30 days through drinking water bottles (800 mL). Five animals from each cage drank an average of 400 mL/day and no significant differences in consumption were observed among the experimental groups. Every week during the 30-day treatment period, the PST concentration (3 or 9 μg/L STX equivalents) in the drinking water was verified by HPLC/FLD. Each rat drank 0.24 or 0.72 μg of PST/day of 3 or 9 μg/L STX equivalents during treatment. PST concentrations in the bottles remained constant for at least a week. Only and when it was necessary, concentration correction to 3 or 9 μg/L was made with dilutions containing growth medium.

The effects of PST or *Aphanothece* sp. on behavior was determined immediately after the 30-day treatment period using the open field habituation task (OFH), the elevated plus maze anxiety (EPM) task, the inhibitory avoidance (IA) task, and the Morris water maze (MWM) task [35–38].

The experimental design was composed of five groups: (1) rats that received BGN/2 medium (control group for *Aphanothece* sp.), (2) rats that received *Aphanothece* sp. (a non-toxic culture), (3) rats that received ASM-1 medium (control group for *C. raciborskii*), (4) rats that received 3 μg/L STX equivalents from PST-producing *C. raciborskii* culture, and (5) rats that received 9 μg/L STX equivalents from PST-producing *C. raciborskii*. Solutions containing the abovementioned five media were renewed daily. Toxin concentrations were also verified every week by HPLC/FLD.

### Behavioral procedures

The OFH task was conducted using a 50 cm high, 60 cm × 40 cm plywood box with a frontal glass wall and a linoleum floor divided in 12 equal rectangles. Animals were left for 5 min both in the training and test sessions, and the number of rearings and crossings between sectors were registered. The difference between the two sessions in the number of rearings and of crossings between rectangles was considered a measure of habituation to the open field. If the animals habituated to the field during the first session, they should recognize it as familiar, and consequently, the number of rearings and crossings should decrease in the second session. The number of crossings in the test session was used as a control for the possible motor and general performance effects of the treatments [35].

The EPM task was conducted using wooden arms that were 50 cm long and 10 cm wide. The apparatus was elevated at a height of 70 cm above the floor. The closed arms were surrounded by a 15 cm wall. Environmental temperature was maintained at that measured in the housing room. The testing room was illuminated with a dim white light that provided 100 lux for the open arms and 50 lux for the closed ones. Each animal, experimentally naive, was placed in the central platform of EPM, facing an open arm, and allowed to freely explore for 5 min. The time spent in the open arms and in the closed arms was registered. After each observation, the apparatus was cleaned with ethyl alcohol (10 %) to remove organic residues left from the preceding animal. Experiments were recorded using a video camera and video files were stored in a personal computer for analyses. The time spent at each open or closed arm was the anxiety index of the task. Rats prefer to stay at hidden places, and thus, they spent more time at the closed arms [36].

The IA task was conducted in an automatically operated and brightly illuminated box in which the left extreme of the grid (42.0 cm × 25.0 cm grid of parallel 0.1 cm caliber stainless steel bars spaced 1.0 cm apart) was covered by a 7.0 cm wide and 5.0 cm high Formica® laminate covered platform. Animals were placed on the platform and their latency to step down with their four paws on the grid was measured. In the training session, immediately upon stepping down, the animals received a 0.5 mA, 3.0 s scrambled footshock. In the test session, no footshock was given and a ceiling of 180 s was imposed to the step-down latency. The latency to step down to the platform in the test session was the aversive memory index for the task [37, 39–42].

The water maze was a black circular pool (190 cm in diameter) conceptually divided into four equal imaginary quadrants for the intention of data analysis. The water temperature was 21–23 °C. Two centimeters below the surface of the water was a black circular platform (12 cm in diameter) hidden from the rats view. It had a rough surface, which allowed the rat to climb onto it easily once its presence was detected. The swimming path of the rats was recorded, in the test session, using a video camera mounted above the center of the pool and analyzed using a video tracking and analysis system. The water maze was located in a white room with several visual stimuli leaned on the walls to provide spatial cues. Rats were handled 5 min per day for three days prior to training. Training in the hidden platform (spatial) version of MWM was conducted during four consecutive days. On each day, rats received four consecutive training trials during which the hidden platform was kept at a constant location. A different starting location was used on each trial, which consisted of swimming followed by sitting during 30 s on the platform. Any rat that did not find the platform within 60 s (time of each trial) was guided to it by the experimenter. The interval between trials was 30 s. During each interval, rats were carefully dried with a towel. Memory retention was evaluated in a 60-s test probe conducted in the absence of the escape platform 24 h after the last training session. In the test probe of MWM, the time spent at the target platform quadrant (TPQ) and at the opposite platform quadrant (OPQ) was registered. The memory retention of the MWM was observed in the probe test session when rats spend more time in TPQ than in OPQ [35, 43].

Statistical analysis was conducted using the data of the behavioral models and included number of rearings and crossings in OFH, latencies to step down in IA, time spent in the close and open arms of EPM, time to arrive at the ridden platform in the trainings of MWM. All data were evaluated by SPSS 18.0 for Windows.

Since the step-down latencies have not passed a normality test (Kolmogorov-Smirnov test with Lilliefors’ correction), differences among groups were evaluated by Kruskal-Wallis ANOVA with Dunn’s all pair-wise multiple comparison *post-hoc* test; training versus test latencies were correspondingly compared by the Wilcoxon signed ranks test. In the OFH, EPM and MWM tasks, as in their respective deviations, variables were normally distributed. Groups were compared by Student’s t-test or ANOVA. In the MWM task, the learning phase was evaluated using repeated measures ANOVA. Significance was set at *p* < 0.05.

## Results

There were no clinical signs of dysfunction in the treated rats throughout the experimental period nor were there any significant deviations in body weight gain in both cyanobacteria treated rats, compared with the controls.

**Fig. 2:**
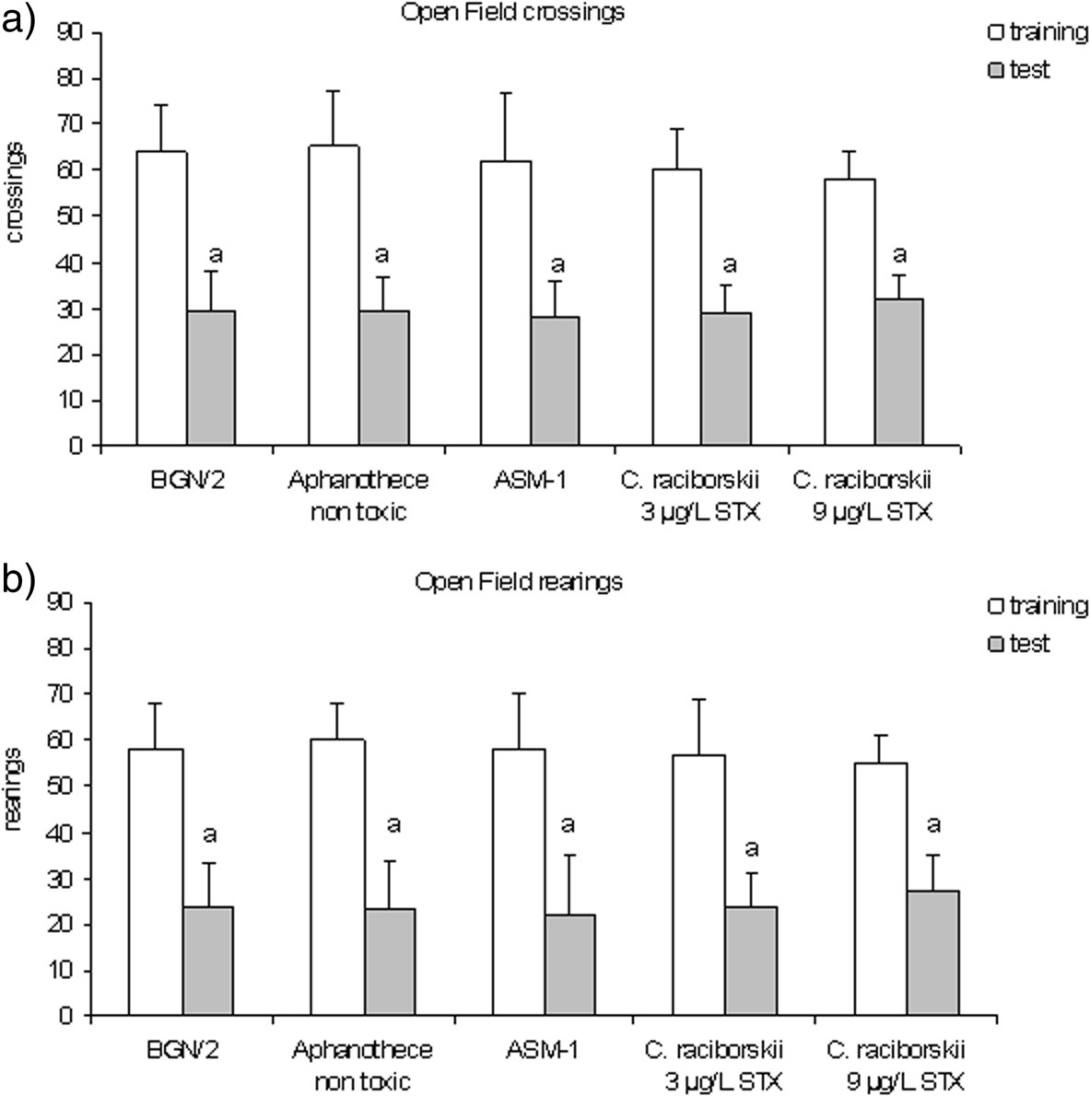
**a** and **b** Absence of effect of all treatments in the OFH task. Data expressed as mean ± SEM; *n* = 10 per group. **a** Number of crossings and rearings in the test probe are significantly different from the corresponding training values for all groups (ANOVA test, *p* < 0.05)

In the OFH (Fig. 2) task, rats received water artificially contaminated with PST-producing *C. raciborskii* for 30 days. None of the experimental groups showed significant differences in the number of rearings or crossings neither in the training nor in the test sessions (*p* > 0.05, one-way ANOVA test). Both variables (rearings and crossings) were significantly lower in the test than in the training session for all groups (*p* < 0.05, ANOVA test), indicating no mnemonic effects on this task. The fact that there were no observed differences in behavior among groups suggests that neither locomotor activity nor exploratory effects have been caused by the chronic consumption of STX-contaminated water (3 or 9 μg/L STX equivalents) or *Aphanothece* sp.

**Fig. 3:**
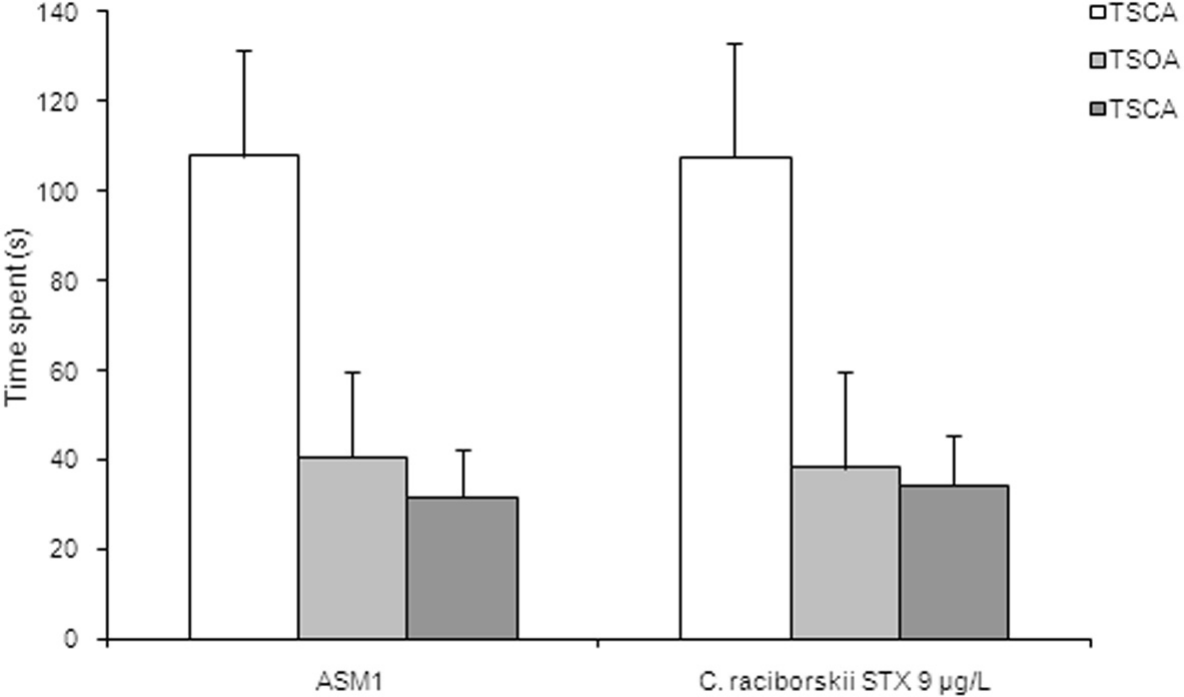
Absence of effect of PST-producing *C. raciborskii* (9 μg/L STX equivalents) in the EPM task. Data expressed as mean ± SEM; *n* = 10 per group; TSCA: time spent at closed arms; TSOA: time spent at open arms; TSCA: time spent at central area

The EPM task (Fig. 3) is an anxiety model. In this experiment, two experimental groups received treatment for 30 days: a control group (ASM1 medium without cyanobacteria) and a PST-producing *C. raciborskii* (9 μg/L STX equivalents) group. Compared to the control group, the STX-treated rats did not show a significant difference in the time spent in the open or closed arms of the EPM apparatus (*p* > 0.05 for both variables, Student’s t-test). These results indicated that neither anxiogenic nor anxiolitic effects were observed by the chronic consumption of PST-contaminated water (9 μg/L STX equivalents).

**Fig. 4:**
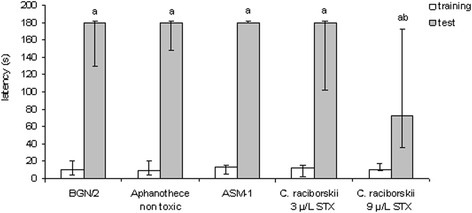
Effect of consumption of cyanobacteria-contaminated drinking water in the step-down IA task. Data expressed as median and interquartile intervals (training session in white; test session in gray); *n* = 10 rats per group. Kruskal-Wallis test shows no significant difference among training session latencies (*p* > 0.05). **a** Each of the five experimental groups has shown a significant difference between training and test session latencies (*p* < 0.05, Wilcoxon test). **b** Only the PST-producing *C. raciborskii* (9 μg/L STX equivalents) group showed a significant difference in the test session latency compared to the control group (*p* < 0.05, Dunn test)

The IA task was a contextual fear conditioning memory model and results are shown in Fig. 4. Five experimental groups were submitted to the test as previously mentioned in the Materials and Methods session. As data were not normally distributed (Kolmogorov-Smirnov normality test, *p* > 0.200), non-parametric tests were used. Only the highest PST concentration (9 μg/L STX equivalents) in drinking water impaired the performance; rats from this group significantly spent lower time at the platform in the test session when compared to the other groups (*p* < 0.05, Dunn’s all pair-wise multiple comparison *post-hoc* test, after a Kruskal-Wallis ANOVA with *p* = 0.011). Groups were comparable because there were no significant differences among training session latencies (*p* = 0.229, Kruskal-Wallis ANOVA). However, all groups have displayed IA task learning, as each test latency was significantly larger than the corresponding training one (*p* < 0.005 for all experimental groups, Wilcoxon signed-ranks test). These results suggested an amnesic effect of the highest concentration (9 μg/L STX equivalents) of long lasting consumption of PST in drinking water.

**Fig. 5:**
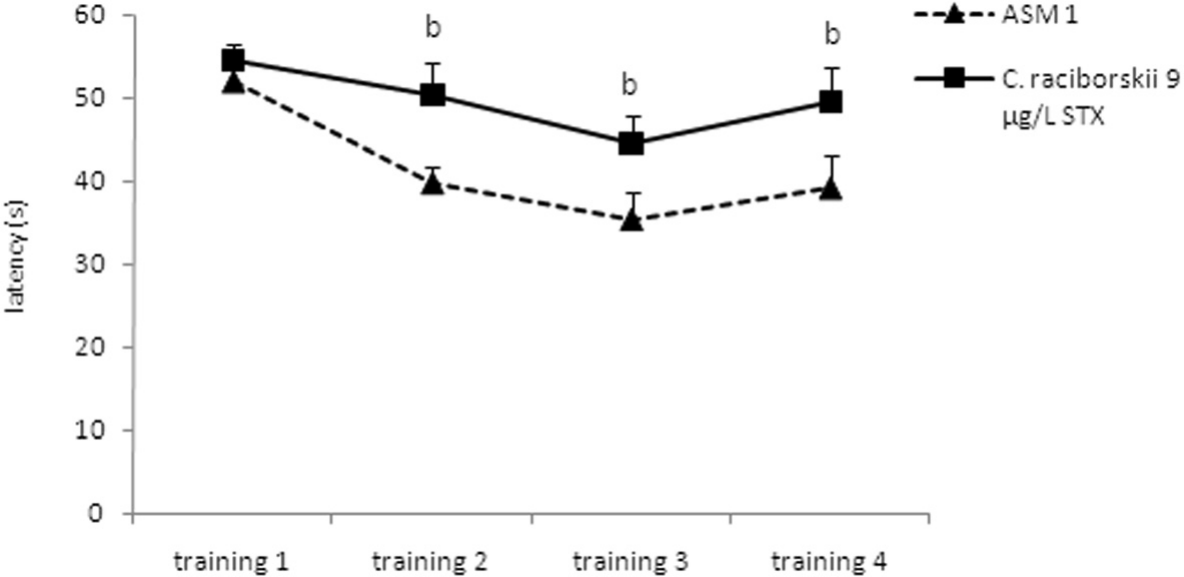
Effect of consumption of cyanobacteria-contaminated drinking water in the training trial of MWM task. Data expressed as mean ± SEM; *n* = 15 rats per group. Both groups showed significant reduction in latency to find the platform during the four training days (*p* < 0.05, repeated measure ANOVA). The PST-producing *C. raciborskii* (9 μg/L STX equivalents) group showed a significant difference compared to the control group (*p* < 0.05, repeated measure ANOVA)

**Fig. 6:**
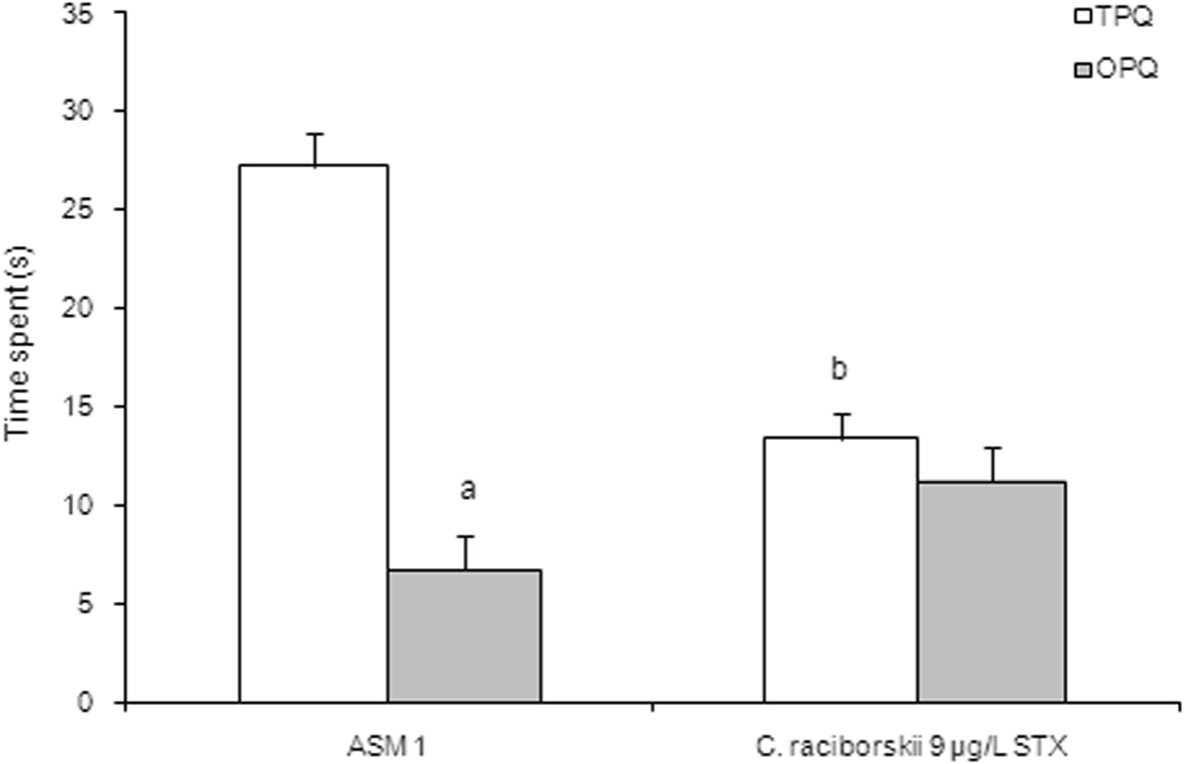
Effect of consumption of cyanobacteria-contaminated drinking water in the test session of MWM task. Data expressed as mean ± SEM; *n* = 15 rats per group. **a** Only the ASM-1 control group showed significant difference between time spent at TPQ and time spent at OPQ, *p* < 0.05 (one-way ANOVA test). **b** Rats treated with PST-producing *C. raciborskii* (9 μg/L STX equivalents) showed significant difference in the time spent at TPQ (*p* = 0.001, one-way ANOVA test and Tukey *post-hoc* test)

**Fig. 7:**
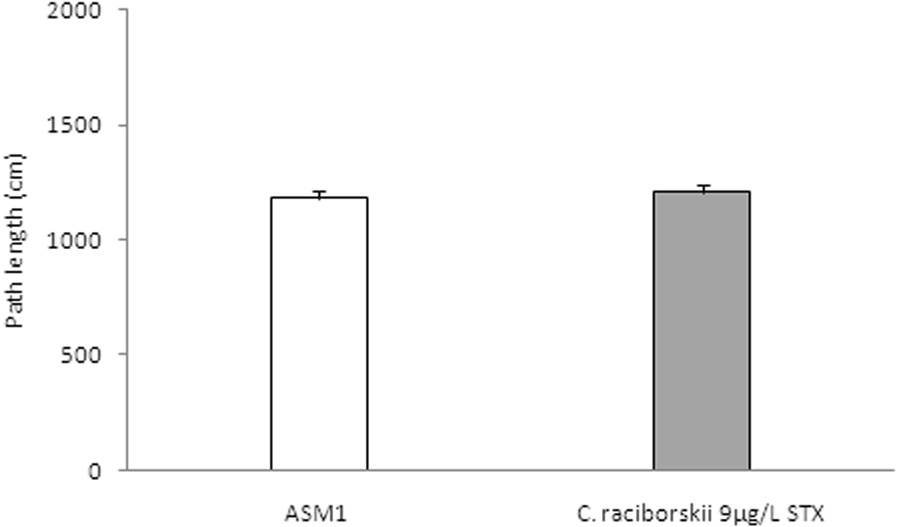
Absence of effect of PST-producing *C. raciborskii* (9 μg/L STX equivalents) on the swimming path length during the test session of the MWM task (*p* = 0.431, one-way ANOVA test). Data expressed as mean ± SEM; *n* = 15 per group

The reference spatial memory model of the MWM task results are shown in Figs. 5, 6 and 7. Two experimental groups were applied: control group (ASM1 medium without cyanobacteria) and PST-producing *C. raciborskii* (9 μg/L STX equivalents).

At the end of the four consecutive training days (Fig. 5), the animals treated with PST took a significantly longer time to find the hidden platform than the control group (*p* = 0.024, repeated measures ANOVA). However, both groups demonstrated a significant latency reduction to find the platform over the four training days (*p* < 0.05, repeated measures ANOVA), indicating learning in both groups.

In the probe test session of the MWM task (Fig. 6), the PST-treated group of rats significantly spent less time at the TPQ than the control group (*p* = 0.001, one-way ANOVA test and Tukey *post-hoc* test). Moreover, the PST-treated group did not show any difference in the time spent at the TPQ compared with the time spent at the OPQ (*p* = 0.218, repeated measures ANOVA). In the test session, there was no significant difference in the path length (Fig. 7) between groups (*p* = 0.431, one-way ANOVA test). Moreover, there was no significant difference between groups concerning swimming speed (*p* = 0.387, one-way ANOVA test), which indicated no motor effects of STX on MWM.

These results suggest an evident amnesic effect of consumption PST-producing *C. raciborskii* in drinking water (9 μg/L STX equivalents) on the spatial reference memory of the MWM task and on the aversive memory of the IA task.

## Discussion

Behavioral alteration could be a reflection of environmental perturbation [44]. Since behavior associates physiological function with ecological processes for a given species, it might provide a useful indicator or biomarker for detecting harmful chemical pollutants [45].

Behavioral changes are a result of a complex modulation in the biochemistry and physiological lower organization levels. The knowledge concerning these fundamental processes is important to understand how an organism would adjust its responses to environmental stimuli. Moreover, adaptation to the environment is a neuronal signal transduction-dependent process in response to biochemistry and genetic expression changes [46].

This study showed results of the effects of long lasting consumption of PST-contaminated drinking water on behavioral parameters and correlated an STX decrease effect to learning and memory processes. In our behavioral study, there was an evident amnesic effect of chronic PST (9.0 μg/L STX equivalents) administration through artificially contaminated drinking water. Memory impairment occurred in both tasks that present different cognitive components: the aversive memory of the IA task, which is dependent on stress modulation (glucocorticoids and noradrenergic activation) [26, 47], and the reference spatial memory of the MWM task [37, 43]. Both memory tasks depend on neurotransmitter system components (glutamatergic, gabaergic and cholinergic), molecular pathways (second messengers and kinases proteins), and brain regions (hippocampal formation, amygdaloid complex and neocortex) related with declarative memory formation in humans [46, 48, 49].

However, no effect was found in the less aversive exploratory OFH task (Fig. 2). The unaltered number of crossings in the OFH task and the effective concentration in the IA and MWM task supports the idea that the effect of PST in the IA and MWM tasks is basically cognitive and not a motor or an exploratory effect [35]. Moreover, in the EPM task, no effect of 9 μg/L STX equivalents was observed, indicating either anxiolytic or anxiogenic status caused by PST [36].

Our non-toxic culture control treatment did not show any effect in OFH and IA tasks. These results supported the idea of a mnemonic effect of PST produced by *C. raciborskii*. However, nonspecific synergy effects caused by secondary metabolites may also contribute with STX in the memory impairment observed.

Furthermore, no effects were observed in the groups treated with 3 μg/L STX equivalents. This result supports the STX concentration guideline in drinking water present in the regulation number 2914/2011 of the Brazilian Ministry of Health. However, recent work reported cytotoxicity, genotoxicity and oxidative stress caused by 3 μg/L of STX in teleost neurons in vitro [50].

Voltage-gated sodium channels, blocked by STX, are expressed for all regions of the vertebrate brain [4, 51]. These channels positively modulate the mechanisms of synaptic plasticity processes and induce long-term potentiation (LTP, memory electrophysiological model) at hippocampus [52, 53]. Our results suggest that STX-sensitive voltage-gated sodium channels positively modulate the learning and memory processes of the IA and MWM tasks.

Co-expression of STX-sensitive voltage-gated sodium channel and receptor tyrosine kinase TrkB at the same places on neurons was reported [51, 53]. Binding of the brain-derived neurotrophic factor (BDNF) to the TrkB receptors co-activates the voltage-gated sodium channel producing rapid neuronal depolarization that induces LTP [51, 53]. Moreover, STX administration over neurons blocks voltage-gated sodium channel and inhibits LTP induced by BDNF [51]. Neurotrophins like BDNF are regulatory factors that mediate differentiation and survival of neurons in the peripheral and central nervous systems and positively modulate learning and memory processes [54].

It was reported that fish exposed to transport stress in the presence of STX displayed significant reduction in neural c-Fos expression [55]. The immediately early gene c-fos, and its protein c-Fos, is known to be induced in neurons of mammals and fish as a result of neuronal stimulation. Moreover, in rats it has been demonstrated that c-Fos expression mediates neuronal excitation and enhances survival, processes associated with memory consolidation [56].

The studies demonstrated that STX impaired memory. The toxic mechanisms of STX on memory processes must be due to damage of the molecular mechanisms involved with control of synaptic plasticity like signaling pathways of BDNF and c-Fos.

However, STX must be involved with oxidative stress and cellular death in some brain regions like hippocampus or neocotex [50, 57]. Recently, it were reported that the consumption of water contaminated with cyanobacteria PSP-producing *Cylindrospermopsis raciborskii* for 30 days caused significant alterations in the antioxidant machinery, both in the brain structures such as the hippocampal formation and the prefrontal cortex as well as in the liver of exposed rats [58]. Thus, memory impairment observed in this work can to be consequence of oxidative stress at hippocampus and prefrontal cortex of rats repeated exposed to saxitoxin-contaminated drinking water.

## Conclusions

In summary, the repeated exposure of rats to contaminated drinking water with *C. raciborskii* producer of PST for 30 days caused deleterious behavioral alterations including memory impairment in inhibitory avoidance task and referential water maze task. The neurotoxic effect of saxitoxin or variants and possible nonspecific damages caused by *C. raciborskii* bloom are problems that require further investigation in order to mitigate a possible impact on the environment.

## Ethics approval

Animal tests were performed in accordance with the guidelines of the Brazilian College of Animal Experimentation (COBEA), and were approved by the Ethics Committee of FURG (process number P025/2011 CEUA/FURG).
